# Lewis y Regulate Cell Cycle Related Factors in Ovarian Carcinoma Cell RMG-I *in Vitro* via ERK and Akt Signaling Pathways

**DOI:** 10.3390/ijms13010828

**Published:** 2012-01-16

**Authors:** Dawo Liu, Juanjuan Liu, Bei Lin, Shuice Liu, Rui Hou, Yingying Hao, Qing Liu, Shulan Zhang, Masao Iwamori

**Affiliations:** 1 Department of Obstetrics and Gynecology, Shengjing Hospital Affiliated to China Medical University, Shenyang 110004, China; E-Mails: cyldw2007@163.com(D.L.); juanjuanliu_lg@yahoo.com.cn (J.L.); liushuicc@sina.com (S.L.); hour@126.com(R.H.); haoyy@163.com(Y.H.); qingLiu@126.com (Q.L.); zhangshl99@126.com (S.Z.); 2 Department of Biochemistry, Faculty of Science and Technology, Kinki University, 3-4-1 Kowakae, Higashiosaka, Osaka 577-8502, Japan; E-Mail: iwamori@163.com

**Keywords:** Lewis(y) antigen, cell cycle, cyclin, cyclin-dependent kinases, cyclin-dependent kinase inhibitors

## Abstract

**Objective:**

To investigate the effect of Lewis y overexpression on the expression of proliferation-related factors in ovarian cancer cells.

**Methods:**

mRNA levels of cyclins, CDKs, and CKIs were measured in cells before and after transfection with the α1,2-fucosyltransferase gene by real-time PCR, and protein levels of cyclins, CDKs and CKIs were determined in cells before and after gene transfection by Western blot.

**Results:**

Lewis y overexpression led to an increase in both mRNA and protein expression levels of cyclin A, cyclin D1 and cyclin E in ovarian cancer cells, decrease in both mRNA and protein expression levels of p16 and p21, and decrease of p27 at only the protein expression level without change in its mRNA level. There were no differences in proteins and the mRNA levels of CDK2, CDK4 and CDK6 before and after gene transfection. Anti-Lewis y antibody, ERK and PI3K pathway inhibitors PD98059 and LY294002 reduced the difference in cyclin and CKI expression caused by Lewis y overexpression.

**Conclusion:**

Lewis y regulates the expression of cell cycle-related factors through ERK/MAPK and PI3K/Akt signaling pathways to promote cell proliferation.

## 1. Introduction

Lewis y is a difucosylated oligosaccharide present on the surface of cells, and its expression is increased in many tumors. Lewis y can regulate gene and/or protein expression through glycosylation modification of cell surface receptors, consequently affecting downstream intracellular signaling pathways. α1,2-fucosyltransferase (α1,2-FT) is a key enzyme in the synthesis of Lewis y. In our previous study, human α1,2-FT was transfected into the ovarian cancer cell line RMG-I by gene transfection technology, and the ovarian cancer cell line RMG-IH with stable and high expression of Lewis y was established. Thus, it was found that Lewis y overexpression accelerates cell entry into S phase from the G0-G1 phase, shortens the cell cycle and enhances cell proliferation [1].

The control of the cell cycle is closely related to tumor cell proliferation, differentiation and apoptosis. Abnormal cell cycle regulation is an important mechanism of tumorigenesis. Cell cycle progression is coordinately controlled by many protein factors, which mainly include cyclins, cyclin-dependent kinases (CDKs), cyclin-dependent kinase inhibitors (CKIs), *etc*. The key to cell cycle regulation is CDK activation during specific cell cycle changes, and the phasic activation of CDKs depends on cyclins. The activities of CDKs can also be inhibited by CKIs. CKIs can exert their functions by binding to CDK alone, or by binding to cyclin-CDK complexes. Two kinds of CKI families have been found so far: one is the INK4 (an inhibitor of CDK4) protein family, including p15, p16, p18 and p19, where each of them contains four ankyrin repeats in its protein structure, mainly specifically inhibiting the phosphorylation kinase activity of the cyclin D1-CDK4/6 complex; the other one is the CIP/KIP protein family, mainly including p21, p27 and p57, which have a nuclear localization signal (NLS) at the *C*-terminus, and a conserved region proximal to the amino-terminus. This group of proteins can exert their inhibitory activities by their interactions with a variety of cyclin-CDK complexes, and thereby induce S phase arrest [2–4].

In our previous study, we had found that Lewis y is a carbohydrate structure of some cell surface receptors, such as EGFR [5], IGF-1R [6], integrin α5 and β1, *etc*. [7], and its overexpression can further activate signaling molecules including Akt and ERK, and then activate the downstream ERK/MAPK and PI3K/Akt signaling pathways [8]. It has been reported that the activation of ERK1/2 affects cell proliferation, apoptosis and migration [9–11]. PI3K/Akt is also considered to be critical in cell proliferation and differentiation [12–14]. The anticancer drug pemetrexed reduces Cdk2 and cyclin-A expression by inhibiting ERK activity, and inhibits the activity of the complex [15]. Chen *et al*. [16] confirmed *in vivo* and *in vitro* that formononetin, an important component of anti-cancer drugs, inhibits the expression of cyclin D1 through the IGF1/PI3K/Akt pathway.

Therefore, we speculated that the acceleration of cell growth induced by Lewis y overexpression may be related to changes in the expression of cell cycle-related factors resulting from activation of the ERK/MAPK and PI3K/Akt signaling pathways. On the basis of preliminary work, this study further investigated the relevant molecular mechanisms of accelerated cell proliferation after overexpression of Lewis y in RMG-I cells, including the effects of its expression on cyclins, cyclin-dependent kinases, protein and mRNA expression status of their inhibitors and corresponding signaling pathways. This study revealed the molecular basis of cell cycle regulation, including that Lewis y overexpression accelerated the proliferation rate of ovarian cancer cells, reduced the proportion of G0/G1-phase cells and increased the proportion of S-phase cells.

## 2. Results

### 2.1. Lewis Y Overexpression Promoted Ovarian Cancer Cells to Enter S Phase

**Figure 1:**
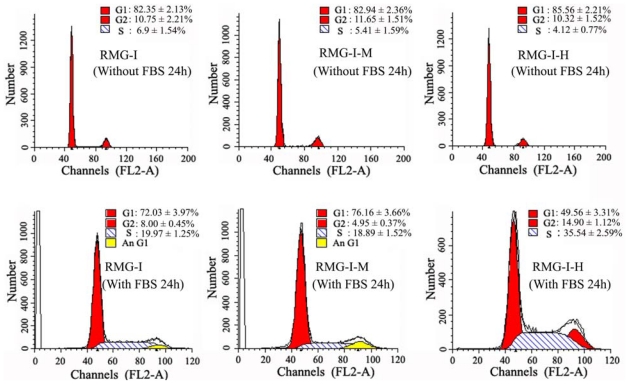
Lewis y overexpression increases the proliferation of RMG-I cells. Cell cycle analysis. RMG-I-M: RMG-I cells transfected with pcDNA3.1 vector; RMG-I-H: RMG-I cells with high expression of the transfected pcDNA3.1/FUT1. Cells were prepared, stained with PI and analyzed by a FAC Scan flow cytometer.

The percentage of RMG-IH cells in G1-phase after gene transfection were significantly reduced compared to either untransfected RMG-I or empty vector-transfected RMG-IM (all *p* < 0.05), while the corresponding percentages of cells in S and G2 phases were significantly increased. These results suggested that Lewis y overexpression, induced by α1,2-fucosyl-transferase gene transfection, promoted RMG-I cell proliferation by altering cell cycle regulation and increasing cell division (Figure 1).

### 2.2. Lewis Y Overexpression Increased mRNA Expression Levels of Cyclins, p16 and p21 Without Affecting Both CDKs and p27 mRNA Expression in Ovarian Cancer Cells

**Figure 2:**
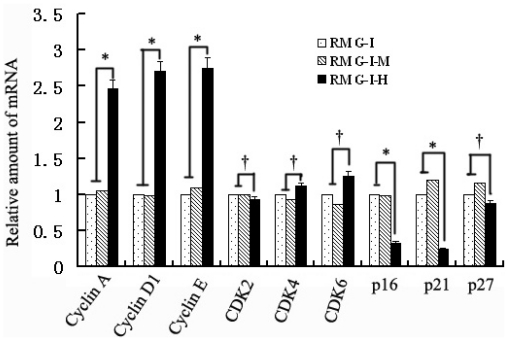
The mRNA expression of Cyclins, cyclin-dependent kinases (CDKs) and cyclin-dependent kinase inhibitors (CKIs) in RMG-I, RMG-I-M, RMG-I-H cells were tested by quantitative Real-Time PCR. RMG-I, RMG-I-M, RMG-I-H: same as Figure 1. Three independent experiments were performed and the results were reproducible. (* *p* < 0.05; ^†^ *p* > 0.05).

Cyclins, CDKs and CKIs all play important roles in the cell cycle, so cell cycle factors closely related to G1/S phases were detected by the real-time PCR method. It was found that mRNA expression levels of cyclin A, cyclin D1 and cyclin E increased in Lewis y overexpressed cells, which were 2.46, 2.71, and 2.75 times those in cells before transfection (all *p* < 0.05), while mRNA expression levels of p16 and p21 in transfected cells were respectively 33.5% and 25.2% of those of cells before transfection with both significantly decreased (both *p* < 0.05). In addition, p27 mRNA levels after transfection tended to decrease, being 87.8% of that before transfection (*p* > 0.05); CDK2, CDK4 and CDK6 expression did not change obviously, which compared to pre-transfection was 92.7%, 1.11 times and 1.26 times, respectively (*p* > 0.05). The results indicated that Lewis y overexpression affected the expression of cyclins and p16 and p21 at the gene level (Figure 2).

### 2.3. Lewis Y Overexpression Promoted Cyclin and CKI Expression Without Affecting CDK Expression in Ovarian Cancer Cells

**Figure 3:**
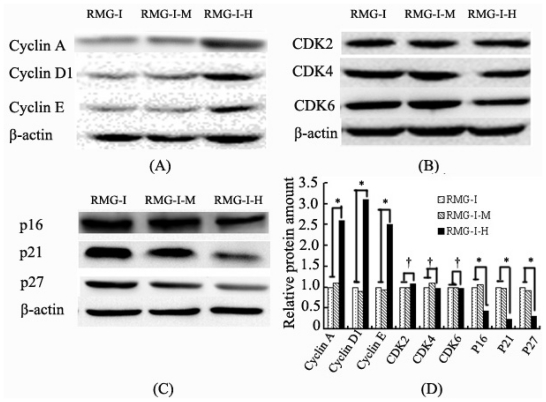
The expression of Cyclins (**A**), CDKs (**B**) and CKIs (**C**) in RMG-I, RMG-I-M, RMG-I-H cells were tested by Western blot; (**D**) Quantification of A, B and C. RMG-I, RMG-I-M, RMG-I-H: same as Figure 1. Three independent experiments were performed and the results were reproducible. (* *p* < 0.05; ^†^ *p* > 0.05).

The protein expression levels of cyclins (cyclins A, D1 and E), CDKs (CDK2, CDK4 and CDK6) and CKIs (p16, p21 and p27) were determined by Western blotting. The results showed that the protein expression levels of cyclin A, cyclin D1, and cyclin E were consistent with their mRNA levels in the transfected RMG-IH cells, which were 2.6, 3.1 and 2.5 times those in the untransfected cells (all *p* < 0.05). Meanwhile, the protein expression levels of p16, p21 and p27 were similar to their mRNA levels, which were significantly reduced to 44%, 23% and 31% of those prior to transfection (all *p* < 0.05), and the protein expression levels of CDK2, CDK4, and CDK6 were 1.09 times, 98% and 97% compared to those in the untransfected cells, consistent with their gene expression levels, *i.e.*, without significant change after transfection (*p* > 0.05) (Figure 3).

### 2.4. Lewis Y Antibody Blocking Reduced Lewis Y Overexpression-Mediated Differences in Cyclin and CKI Protein Expression

Lewis y antibody was used to conduct a blocking study in Lewis y-overexpressed cells. The antibody block the activation of Lewis y and interfering with the dynamics and signaling of EGFR or some other cell surface receptor which carbohydrate structure contained Lewis y. The results showed that the protein expression levels of cyclin A, cyclin D1 and cyclin E were reduced in the treated cells compared to those in the cells without blocking treatment (all *p* < 0.05), whereas p16, p21 and p27 protein levels increased (all *p* < 0.05), and the expression differences relative to the untransfected cells were significantly reduced (Figure 3).

### 2.5. The Differences in Cyclin and CKI Protein Expression Levels Were Decreased by Treatment with LY294002 and PD98059 in Lewis Y Overexpressed Ovarian Cancer Cells

**Figure 4:**
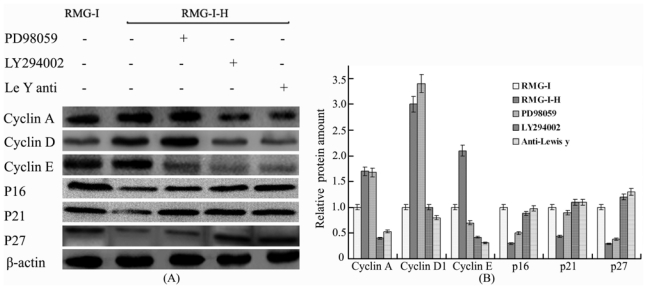
Effect of the ERK, PI3K/Akt signaling pathways and Lewis y antibody on Lewis y induced expression of Cyclins and CKIs protein expression. (**A**) Western blot profiles of Cyclins and CKIs. RMG-I, RMG-I-H: same as Figure 1.RMG-I-H cells were pretreated with PD98059 (25 μM), LY294002 (50 μM), or anti-Lewis y antibody (10 μg/mL) for 24 h; (**B**) Quantification of A. Data were expressed as the mean ± SD from three independent experiments.

The ERK/MAPK pathway inhibitor PD98059, PI3K/Akt pathway inhibitor LY294002 and DMSO were used to treat Lewis y-overexpressed cells, respectively. The results showed that the differences in the proteins expression levels of cyclin A, cyclin D1, cyclin E, p16, p21 and p27 before and after treatment were also apparently reduced in the cells (Figure 4).

## 3. Discussion

The progression of the cell cycle is regulated by many factors. There are two main regulation points: one at the G1/S restriction point, which controls cells from entering the S phase, and the other at the G2/M turning point. Non-proliferative cells are in the G0 phase, and when stimulated by growth factors, *etc*., they can enter the S phase through the G1/S restriction point, exiting the G1 phase. In mammalian cell cycle regulation, the regulation points between G1-S phase are key points for the control of cell proliferation. Abnormalities in these regulation points are closely related to the formation and development of tumors. Our previous work had found that Lewis y overexpression significantly reduces ovarian cancer cell number in G0-G1 phase, and significantly increases the percentage of cells in S and G2 phases compared to that in untransfected cells [1], indicating that the cell cycle is in a proliferative state. In this study, we found that the mRNA and protein expression levels of cyclin A, cyclin D1 and cyclin E significantly increased in Lewis y-overexpressed cells and that the mRNA and protein expression levels of CDK2, CDK4 and CDK6 did not change. Cyclin A, E and D1 are positive regulatory proteins that promote G1/S transition in the cell cycle, and CDKs are the core of the cell cycle regulatory network. Different cyclin proteins bind to their corresponding CDKs and form corresponding cyclin-CDK complexes to activate the activities of CDK kinases, and they are thus involved in cell cycle regulation. By binding to CDK2 to form a complex, cyclin A initiates S-phase DNA replication to complete the S phase, and assists in G2/M phase transition [17]. In our *in vitro* culture system, the cyclin A mRNA and protein expression levels in Lewis y-overexpressed cells significantly increased compared to those in the untransfected cells, which was consistent with the increased proportion of cells in the G2/M phase. Cyclin D1 binds to corresponding CDK4 or CDK6 to form a complex, activates Rb proteins, initiates the transcription of S phase-related genes, and then allows the cells to enter into an autonomous division and proliferation program, resulting in CDK kinase activation and the cell division entering S phase from G1 phase [18]. Currently, cyclin D1 protein overexpression has been found in the majority of tumors (breast, gastric, esophageal and ovarian cancer, and B-cell lymphoma). Cyclin E mainly forms a complex with CDK2 to drive the G1-S phase, initiate DNA synthesis in S phase, and promote cell cycle progression [19]. Gene amplification and protein expression disorder of cyclin E can be found in breast, colon, gastric and ovarian cancer, among others [20]. This study found that the increased mRNA and protein expression levels of cyclin D1 and cyclin E caused by Lewis y overexpression were consistent with the decreased proportion of G1 phase in ovarian cancer cells resulting from Lewis y overexpression. After the cells were treated with anti-Lewis y monoclonal antibody, the mRNA and protein expression levels of cyclin A, cyclin D1 and cyclin E were significantly reduced, which further confirmed our conclusions.

Negative regulatory factors also play important roles in cell cycle control. At present, many studies have shown that p16, p21 and p27 most likely directly act on the regulation of the G1/S phase restriction point [21–23]. P16 can bind to CDK4/6, and the cyclin D1-CDK complex, thereby inhibiting the phosphorylation of Rb proteins and the release of transcription factor E2F, which in turn induces G1-S phase arrest. The P21 protein is an inhibitor that binds to almost every cyclin-CDK complex, and makes the complex lose its kinase activity, which leads to cell cycle arrest in G2 phase. P27 arrests the cell cycle in G1 phase and inhibits cell proliferation by binding to the activated cyclin-CDK2 complex and cyclin-CDK4 complexes and then inhibiting their activities. In this study, it was found that in Lewis y-overexpressed cells, both mRNA and protein expression levels of P16 and P21 were reduced; although reduction in P27 mRNA was not obvious, its protein level was significantly decreased. After the cells were treated with anti-Lewis y monoclonal antibody, the above changes were reversed, which further confirmed that the promotion of G1-S transition by Lewis y was related to the inhibition of p16, p21 and p27.

The synthesis, assembly, modification and activation, disassembly, degradation and conversion of cyclins, CDKs, and CKIs are the core mechanisms that lead to the progression of cell cycle phases and the phase transitions and are regulated by signal transduction from extracellular growth factors. These signaling molecules, including mitogens and anti-mitogens, transduce the corresponding signals downward after binding to their receptors, and ultimately affect the cell cycle through a complex network system. The PI3K/Akt and ERK/MAPK signal transduction pathways are important pathways for cell survival and apoptosis inhibition. Studies have shown that IGF-1 upregulates cyclin D1 and cyclin E expression via the MAPK signaling pathway [24]; p21 is a direct substrate for Akt proteins, and activated Akt phosphorylates the Thr145 site in p21 proteins, which promotes the extranuclear transport of p21, leads to the accumulation of the proteins in cytoplasm, and thus facilitates p21 degradation [25]. TGF-β is an anti-mitogen that can arrest cells in late G1 phase, which is caused by p27 binding to the CDK2-cyclin E complex and inhibiting the activity of the latter [26,27]. The ERK/MAPK and PI3K/Akt signaling pathway inhibitors were used in the transfected cells for corresponding inhibition, and it was found that the differences in cyclin and CKI expression levels were significantly reduced. Combined with our previous findings that Lewis y overexpression leads to a significant increase in phosphorylation levels of Akt and ERK1/2 [5], increase in Lewis y antigen, as a part of the structure of IGF-1R, EGFR and other cell surface receptors [6,8], not only activates the receptor tyrosine kinase activities but also further activates its downstream PI3K/Akt and Raf/MEK/MAPK signaling pathways. We speculate that increased expression of Lewis y antigen accelerates gene transcription in nuclei by activating the above pathways, stimulates DNA synthesis, alters expression levels and activities of cycle-related proteins, and ultimately promotes cells to skip the G1 phase restriction point and enter into S phase for cell proliferation.

The results of this study provide a detailed theoretical basis of how Lewis y overexpression causes accelerated proliferation of ovarian cancer cells. Lewis y regulates the expression of cell cycle-related factors through the ERK/MAPK and PI3K/Akt signaling pathways, thereby affecting cell proliferation.

## 4. Materials and Method

### 4.1. Reagents and Cell Lines

The RMG-I cell line, which was originated from human ovarian clear cell carcinoma, donated by Professor Iwamori Masao of Tokyo University of Japan. *α1,2*-*FT* gene transfected RMG-I cell line was established as previously reported [25], named as RMG-I-H. RMG-I-M was the cell line transfected with the vector alone. The following reagents were purchased from commercial sources: DMEM and fetal bovine serum (FBS) from Hyclone (Logan, UT, USA); trypsin and ethylenediamine tetraacetic acid (EDTA) from Amresco; Mouse anti-human Lewis(y) mAb (clone A70-C/C8) was purchased from Abcam (England). The anti-p16, p21, p27 and β-actin were purchased from Santa Cruz (USA). The anti-Cyclin A and Cyclin D1 were purchased from Bioworld (USA). The anti-Cyclin E and CDK2 were purchased from Epitomics (USA). The anti-CDK4 and CDK6 were purchased from Thermo (USA). Ly294002 and PD98059 were obtained from Sigma (USA). Trizol reagent, PrimeScript™ RT reagent kit, and SYBR^®^ Premis Ex Taq™ from TaKaRa Biotechnology Co. (China). The sequences of primers were synthesized by Invitrogen Co. (China).

### 4.2. Cell Culture

Cells were cultured in DMEM supplemented with 10% FBS at 37 °C, 5% CO_2_ in humidified air. Cells are grouped in western blot as follows: Lewis y antibody-treated groups (cells were treated with 10 μg/mL Lewis y monoclonal antibody at 37 °C in 5% CO_2_ for 24 h), irrelevant isotype-matched control (10 μg/mL normal mouse IgM), PD98059-treated goups (25 μM at 37 °C in 5% CO_2_ for 24 h), LY294002-treated goups (50 μM at 37 °C in 5% CO_2_ for 24 h).

### 4.3. Analysis the Effect of Lewis Y Antigen on Cell Proliferation

A total of 1 × 10^5^ cells in 1 mL were inoculated into a 6-well plate in serum-free medium, with each group in triplicate, and cultivated for 24 h. The cells were synchronized in G1 phase and then further cultivated in medium with 10% serum for 24 h. The three groups of cells were collected, washed twice with PBS, detached with trypsin to prepare a single cell suspension, and centrifuged at 1000 rpm for 5 min. The supernatant was discarded. After being fixed in 70% cold ethanol and stained with PI (propidium iodide) solution, the cells in suspension were submitted to flow cytometry for quantitative cell cycle analysis.

### 4.4. Determination and Real-Time PCR

Total RNA was extracted from the transfected and control cells using Trizol reagent. cDNA was synthesized according to the RNA reverse transcription kit instructions and was used as a template for PCR analysis. Then the cDNA was subjected to Real-time PCR analysis using the SYBR Green PCR Master Mix on the ABI Prism 7500 Sequences Detection System. The PCR primer sequences were designed according to the human Cyclin A, D1, E, *CDK2*, *CDK4*, *CDK6*, *p16*, *p21* and *p27* gene sequences reported in GenBank and chemically synthesized (Table 1). The specificity of the PCR was confirmed by examining the dissociation reaction plot subsequent to Real-time PCR. Human glyceraldehydes-3-phosphate dehydrogenase (GAPDH) served as the constitutive control. PCR reactions of each sample were done in triplicate. Data were analyzed through the comparative threshold cycle (C_T_) method.

### 4.5. Analysis of the Proteins of Cell Cycle Relation Factors with Western Blotting

Cells were washed twice with ice-cold PBS, scraped in lysis buffer (50 mM Tris-HCl (pH 7.4), 150 mM NaCl, 0.5% NP40, 100 mM NaF, 200 μM Na_3_VO_4_, and 10 μg/mL each aprotinin, leupeptin, PMSF, and pepstatin), and incubated for 30 min at 4 °C while rocking. Lysates were cleared by centrifugation (10 min at 12,000 rpm, 4 °C). For immunoblot analysis, 50 μg of total protein were resolved by SDS-PAGE and transferred to poly (vinylidene difluoride) membranes. Membranes were blocked with TTBS (25 mM Tris-HCl, 150 mM NaCl (pH 7.5), and 0.1% Tween 20) containing 5% nonfat milk and incubated overnight at 4 °C with antibody Cyclin A (1:500), Cyclin D1 (1:500), Cyclin E (1:1000), CDK2 (1:1000), CDK4 (1:200), CDK6 (1:200), p16 (1:200), p21 (1:200) and p27 (1:200) in TBST/1% nonfat milk. Blots were washed in TTBS and incubated with the appropriate horseradish peroxidaselinked IgG, and immunoreactive proteins were visualized with ECL detection system. The protein bands were quantified with densitometric analysis. The expression of each protein was calculated by the ratio of the intensity of this protein to that β-actin.

### 4.6. Statistical Analysis

All experiments were performed in triplicate and all data are expressed as mean ± standard errors. Raw data were analyzed by the unpaired Student’s *t* test using SPSS 11.0 software (USA). A *p*-value < 0.05 was considered to be statistically significant.

## 5. Conclusions

Lewis y overexpression increased cell proliferation by regulation cell cycle regulated factors with the cooperation of ERK/MAPK and PI3K/Akt pathways.

## Figures and Tables

**Table 1:** Characteristic of primers used for Real-time PCR amplification.

Gene name	Sequence of primers(5′-3′)	Product length (bp)	Annealing temperature (°C)
Cyclin A	F: AGGTACTGAAGTCCGGGAAC R: GTGACATGCTCATCATTTACAGGAA	106	62
Cyclin D1	F: TGATGCTGGGCACTTCATCTG R: TCCAATCATCCCGAATGAGAGTC	177	63
Cyclin E	F: TTTGCAGGATCCAGATCAAGA R: CACAGACTGCATTATTGTCCCAAG	92	60
*CDK2*	F: CTCCACCGAGACCTTAAACCTCAG R: TCGGTACCACAGGGTCACCA	138	60
*CDK4*	F: CTTCTGCAGTCCACATATGCAACA R: CAACTGGTCGGCTTCAGAGTTTC	114	60
*CDK6*	F: GATCTCTGGAGTGTTGGCTGCATA R: GGCAACATCTCTAGGCCAGTCTTC	144	62
*P16*	F: GGCACCAGAGGCAGTAACCA R: GGACCTTCGGTGACTGATGATCTAA	126	63
*P21*	F: AAGACCATGTGGACCTGTCACTGT R: GAAGATCAGCCGGCGTTTG	155	62
*P27*	F: CAAATGCCGGTTCTGTGGAG R: TCCATTCCATGAAGTCAGCGATA	177	63
